# Acute supplementation with an amino acid mixture suppressed the exercise-induced cortisol response in recreationally active healthy volunteers: a randomized, double-blinded, placebo-controlled crossover study

**DOI:** 10.1186/s12970-020-00369-2

**Published:** 2020-07-23

**Authors:** Yuichi Tsuda, Rika Murakami, Makoto Yamaguchi, Taiichiro Seki

**Affiliations:** 1 R&D Division, Meiji Co., Ltd., 1-29-1 Nanakuni, Hachiouji, Tokyo, 192-0919 Japan; 2 College of Bioresource Sciences, Nihon University, 1866 Kameino, Fujisawa, Kanagawa 252-0880 Japan

**Keywords:** Amino acid mixture, Exercise, Cortisol

## Abstract

**Background:**

Few studies have demonstrated the suppressive effects of amino acids (AAs) on the level of cortisol during exercise in humans. We hypothesized that an AA mixture containing arginine, which promotes lipid metabolism, valine, which effectively decreases the level of glucocorticoid, and serine, a substrate in the production of phosphatidylserine that is reported to blunt increases in cortisol, would suppress the exercise-induced cortisol response by combining the positive effects of the AAs synergistically.

**Methods:**

A randomized, double-blinded, placebo-controlled crossover trial was conducted. Twenty healthy recreationally active males ingested either an AA mixture containing 1.8 g of arginine, 1.1 g of valine, and 0.1 g of serine or a placebo. Thirty minutes after ingestion, subjects performed an exercise trial on a cycle ergometer for 80 min at 50% maximal oxygen consumption. Plasma cortisol and other blood parameters immediately before and after the exercise were evaluated.

**Results:**

Plasma cortisol concentrations after exercise were significantly higher than those before exercise in the placebo condition (9.51 ± 0.85 vs 14.39 ± 2.15, *p* < 0.05), while there was no significant difference in the AA condition (9.71 ± 0.93 vs 9.99 ± 1.23, *p* = 0.846). In addition, the increase in plasma cortisol before and after exercise was significantly lower in the AA condition than in the placebo condition (0.28 [− 2.75, 3.31] vs 4.87 [0.89, 8.86], *p* < 0.05). For the level of adrenocorticotropin, there was a significant difference between before and after exercise only in the placebo condition (24.21 ± 2.91 vs 53.17 ± 6.97, *p* < 0.01) but not in the AA condition (27.33 ± 3.60 vs 46.92 ± 10.41, *p* = 0.057). Blood glucose, plasma lactate, plasma ammonia, serum creatine phosphokinase, serum total ketone body, and serum free fatty acid were also significantly changed by the exercise load in both conditions, but no significant differences were observed between the two conditions.

**Conclusions:**

The present study demonstrated that the AA mixture suppressed the cortisol response during exercise without affecting exercise-related biological parameters such as glucose or lipid metabolism.

**Trial registration:**

UMIN Clinical Trials Registry, UMIN000023587. Registered 19 August 2016.

## Background

Stress causes activation of the hypothalamic-pituitary-adrenal (HPA) axis [1, 2]. The pathway is initiated by the release of corticotropin-releasing hormone (CRH) from the hypothalamus, which then stimulates the anterior pituitary to release adrenocorticotropin (ACTH) into the circulation. ACTH then stimulates the adrenal cortex to release the glucocorticoid cortisol (in humans). Many studies have shown that exercise-induced stress also stimulates the HPA axis and increases cortisol secretion [3–6]. It was also demonstrated that decreases in the blood glucose concentration trigger the pituitary-adrenocortical axis to enhance secretion of ACTH and cortisol during prolonged exercise in humans [7, 8]. Moreover, there were significant correlations between the cortisol response and the sense of fatigue during stressful exercise [9, 10]. These reports point out that cortisol is released in response to glycogen depletion or reduced blood glucose levels to maintain the blood glucose level, delaying fatigue. Luger et al. reported that physical conditioning is associated with a reduction in pituitary-adrenal activation in response to exercise (i.e., highly trained athletes have diminished responses of ACTH and cortisol to CRH) [11]. This indicates that the increase in mitochondrial capacity by training shifts fuels to more fat burning and glycogen sparing, thus preventing reduced blood glucose levels, which can contribute to a reduction in the pituitary-adrenal response. In addition, many studies have shown that cortisol elevation induces protein breakdown [12–15], which might trigger glucocorticoid-induced muscle atrophy [16, 17]. Thus, the suppression of the level of cortisol means less stress or better physical condition (sparing glycogen, maintaining blood glucose level, etc.) during exercise and contributes to the reduction in the post-workout catabolic breakdown of protein.

There are some nutritional strategies for the suppression of cortisol levels during exercise. For instance, several studies have demonstrated that the consumption of carbohydrates during aerobic exercise contributes to reducing postexercise cortisol levels [18–20]. Although there have also been several reports evaluating the effects of amino acid (AA) supplementation on the exercise-induced cortisol response, few studies have elucidated the suppressive effects of amino acids on the level of cortisol during exercise in humans. It was reported that branched-chain amino acid (BCAA) administration before exercise did not affect the response of cortisol, while BCAA administration decreased the growth hormone concentration during exercise and increased the level of testosterone during the recovery period [21]. A powder supplement containing 5.2 g of BCAAs, 4.3 g of essential amino acids, and 1.5 g of taurine did not have a significant effect on the acute cortisol or testosterone response to resistance exercise [22]. On the other hand, some reports indicate that some AAs may have the potential to reduce exercise-induced cortisol. It was reported that arginine administration promoted lipid metabolism in rodents and humans [23–25], which might lead to the maintenance of glucose or glycogen levels during exercise and therefore suppress the cortisol increase. The acute supplementation of valine is reported to effectively decrease the level of glucocorticoid (corticosterone in rodents) by maintaining liver glycogen and blood glucose in a rodent study [26]. Serine is known as a substrate in the production of phosphatidylserine, which is reported to blunt the increases in cortisol levels during exercise [27]. In addition, we demonstrated that an AA mixture containing 1.8 g of arginine, 1.1 g of valine, and 0.1 g of serine supplement could reduce exercise-induced fatigue in a previous study [28]. In that study, however, the AA mixture supplementation did not significantly suppress the cortisol concentration compared to the placebo. A certain number of subjects showed very little change in plasma cortisol concentration during exercise in the study. The exercise condition was probably not enough for them to stimulate the cortisol response. To evaluate the effect of the supplement on the cortisol response, it is important to exclude noncortisol responders. We hypothesized that the above-described AA mixture containing arginine, valine, and serine could suppress exercise-induced cortisol in subjects who have a high cortisol response to the exercise protocol. First, arginine, which promotes lipid metabolism [23–25], might contribute to suppressing the cortisol response by maintaining glucose or glycogen levels during exercise. Second, valine might also act to decrease the cortisol level via the maintenance of glycogen and blood glucose [26]. In addition, serine might blunt the increase in cortisol levels by producing phosphatidylserine, which is a different mechanism from arginine or valine [27].

Here, we conducted a randomized, double-blinded, placebo-controlled crossover study in recreationally active healthy subjects. The aim of the present study was to examine the effect of acute supplementation of the AA mixture on the exercise-induced cortisol response in humans. We hypothesized that AA mixture supplementation would suppress the cortisol response during exercise by combining the positive effects of arginine, valine, and serine synergistically.

## Methods

### Subjects

**Fig. 1:**
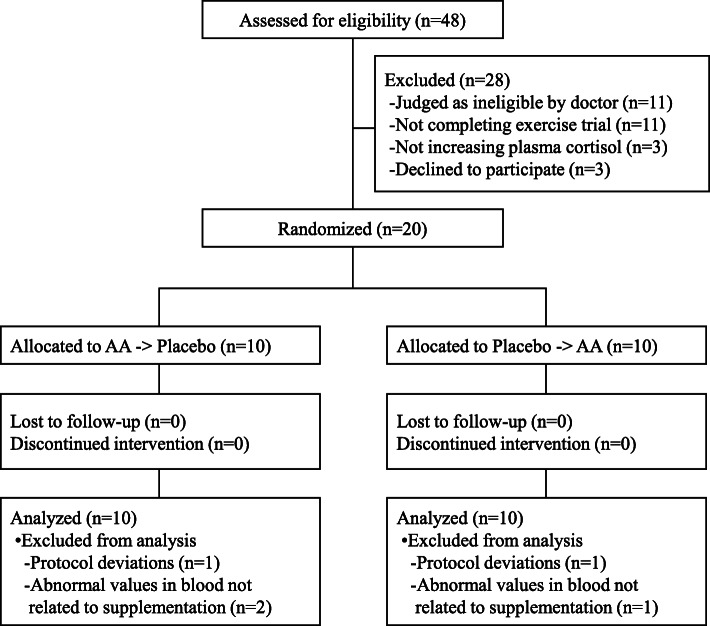
Flowchart of the study participants

Forty-eight recreationally active healthy male volunteers aged 20 to 39 years old were recruited. All study participants provided written informed consent prior to participation in the study. The exclusion criteria consisted of subjects with food allergies or a smoking habit, subjects who had blood samples of more than 200 ml or 400 ml taken within 1 month or 3 months prior to the start of the study, subjects who participated in other clinical studies within the prior month, or subjects who were judged as ineligible by a doctor for other reasons. Subjects who could not complete an exercise trial that was the same as the main trials or subjects whose plasma cortisol did not increase at all by the exercise trial were also excluded. The recruitment and selection of participants were conducted between August and September 2016. A total of 20 subjects were randomly allocated into two groups in a 1:1 ratio using a computer-generated random number sequence by an investigator who had no contact with the subjects or researchers. The sequence allocation concealment and blinding of the subjects and researchers were maintained throughout the study. The sample size of subjects was selected to achieve 80% power at a 5% significance level. To set the sample size, we also referred to other studies that have evaluated nutritional interventions on blood parameters during exercise [21, 22]. In the study, all subjects completed the entire protocol. Before the statistical analysis, three participants were removed due to abnormal values in their blood analyses not related to the supplementation, and two were removed due to protocol deviations. We therefore analyzed a final total of 15 subjects (Fig. 1). The basic characteristics of the study subjects are shown in Table 1. The subjects were instructed not to change their usual exercise volume or diet during the study and not to consume any supplements on the trial day. They were also prohibited from consuming of alcohol on the day before and on the day of the exercise test and instructed to keep their caffeine intake unchanged.

**Table 1:** Basic characteristics of the study subjects

	*n* = 15
Age (years)	32.3 ± 1.2
Height (cm)	169.9 ± 1.5
Body weight (kg)	64.4 ± 1.5
BMI (kg/m^2^)	22.3 ± 0.4
VO_2_ max (mL/min/kg)	39.8 ± 1.7

Values are the mean ± SEM

### Study design

A randomized, double-blinded, placebo-controlled crossover trial was conducted with two groups at the CPCC Company Limited (Tokyo, Japan). First, the individual’s maximal oxygen consumption (VO_2_max) was measured using an incremental cycle exercise test on a cycle ergometer (Aerobike 75XLIII, Konami Sports Life Co., Ltd., Tokyo, Japan). After more than 1 week, participants conducted the exercise trial that was the same as the main trials on a cycle ergometer to determine whether the subjects could complete the exercise trial and whether plasma cortisol increased as a result of the trial. After selection and allocation, the subjects participated in the main experimental trials. On the exercise day, subjects had the same breakfast. After visiting the clinic, subjects ingested cellulose capsules (Matsutani Chemical Industry Co., Ltd., Hyogo, Japan) containing 1.8 g of arginine, 1.1 g of valine, and 0.1 g of serine (Kyowa Hakko Bio Co., Ltd., Tokyo, Japan) or empty cellulose capsules as the placebo 30 min before the exercise trial in a randomized order and in a double-blinded fashion. They were given a cup containing either the AA mixture capsules or the placebo capsules and instructed to take them promptly without directly picking them up, which made them indistinguishable. The blending ratio of AAs followed the previous study [28], and the dose of the AA mixture was also determined according to the previous study [28], which was approximately the same as those in the other studies. Then, they carried out an exercise trial on a cycle ergometer (Aerobike 75XLIII) at 50% VO_2_ max for 80 min with a 3-min rest at the midpoint. To prevent dehydration, subjects drank equal amounts of water during a 3-min rest. The exercise protocol was determined by referring to a past study in which the level of plasma cortisol was increased in untrained men [29]. The exercise trial was conducted in a temperature- and humidity-controlled environment. After more than a 1-week washout period, the participants repeated the same trial with the other test sample. These trials were conducted between September and October 2016. The study protocol was approved by the Institutional Review Board of Chiyoda Paramedical Care Clinic (Tokyo, Japan) (Approval No. 20160720) and the Meiji Institutional Review Board (Tokyo, Japan) (Approval No. 91) and was registered in the UMIN Clinical Trials Registry (UMIN000023587) on August 19, 2016. The study was conducted in accordance with the Declaration of Helsinki.

### Blood sampling

Blood samples were collected from the brachial vein immediately before and after exercise. Whole blood in an EDTA-2Na-containing tube was centrifuged immediately at 1700 *g* for 10 min at 4 °C, and the plasma was separated for analyses of cortisol and ACTH. Serum samples were prepared by collecting whole blood in a plain tube and centrifuging the blood at 1700 *g* for 10 min at 4 °C for analysis of total ketone bodies, free fatty acids, and creatine phosphokinase (CPK). Whole blood was deproteinized in a 1 N perchloric acid-containing tube for 15–60 min on ice and was then centrifuged at 1700 *g* for 10 min at 4 °C for lactate and ammonia analysis. Whole blood collected in a NaF- and EDTA-2Na-containing tube was used for analysis of glucose. Assays of blood parameters were performed at LSI Medience Corporation (Tokyo, Japan).

### Statistical analysis

In the study, the primary outcome was plasma cortisol concentration, and secondary outcomes were the concentrations of plasma ACTH, blood glucose, plasma lactate, plasma ammonia, serum CPK, serum total ketone body, and serum free fatty acid. All data are expressed as the mean ± standard error of the mean (SEM) or the change score with 95% confidence interval (CI). Data were analyzed using a two-way repeated measures analysis of variance (ANOVA) with treatment (placebo and AA) and time (before exercise and after exercise). Significant main effects and interactions were explored with a Bonferroni-corrected post hoc t-test. The change scores before and after exercise were analyzed using Student’s t-test for the comparisons of pairs of conditions. The correlations between the changes in cortisol and ACTH were analyzed using Spearman’s rank correlation analysis. Analyses were performed with SPSS v. 22 (IBM Japan, Ltd., Tokyo). Differences with *p*-values < 0.05 were considered significant.

## Results

No subjects reported any side effects related to the supplementation, and there was no significant treatment order effect in the study.

### Cortisol and ACTH

**Fig. 2:**
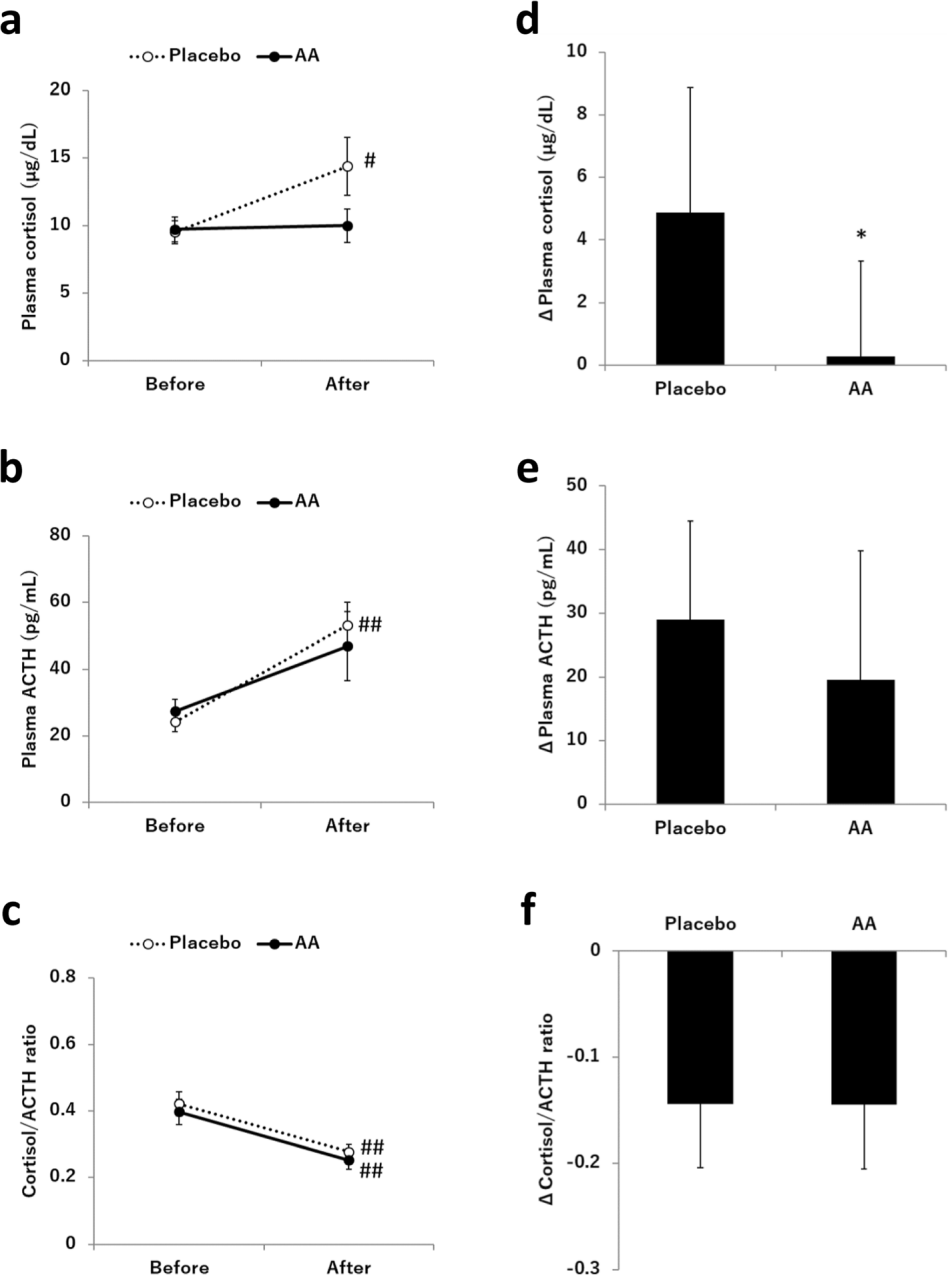
Effects of AA mixture supplementation on plasma cortisol, ACTH, and the cortisol/ACTH ratio. The levels of **a** plasma cortisol, **b** plasma ACTH, and **c** the cortisol/ACTH ratio before and after exercise are shown. In addition, the changes in **d** plasma cortisol, **e** plasma ACTH, and **f** the cortisol/ACTH ratio before and after exercise are shown. Values are the mean ± SEM or change score with 95% CI (*n* = 15). # *p* < 0.05, ## *p* < 0.01 compared to before exercise. * *p* < 0.05 compared to the placebo

**Fig. 3:**
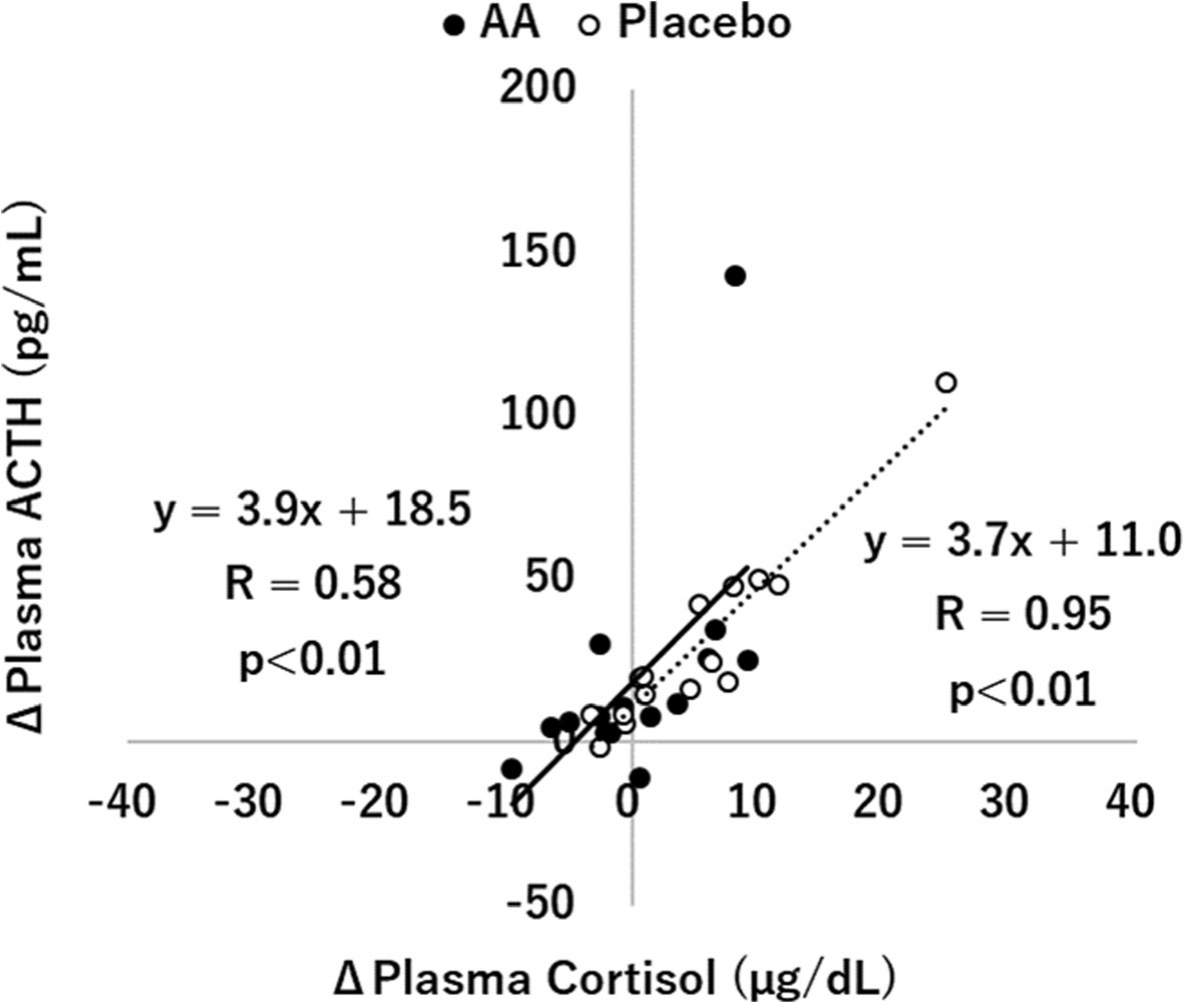
Correlations between the change in plasma cortisol and ACTH in the AA and placebo conditions

The effects of supplementation with the AA mixture on plasma cortisol, ACTH, and the cortisol/ACTH ratio are shown in Fig. 2. A two-way repeated measures ANOVA (treatment × time) for plasma cortisol revealed a significant interaction between treatment and time (*p* < 0.05). Post hoc t-tests revealed that the level of plasma cortisol after exercise was significantly higher than that before exercise in the placebo condition (9.51 ± 0.85 vs 14.39 ± 2.15, *p* < 0.05), while there was no significant difference in the AA condition (9.71 ± 0.93 vs 9.99 ± 1.23, *p* = 0.846). In addition, the increase in plasma cortisol before and after exercise was significantly lower in the AA condition than in the placebo condition (0.28 [− 2.75, 3.31] vs 4.87 [0.89, 8.86], *p* < 0.05). ANOVA for plasma ACTH showed that there was a significant main effect for time (*p* < 0.01), with post hoc t-tests revealing a significant difference between before and after exercise only in the placebo condition (24.21 ± 2.91 vs 53.17 ± 6.97, *p* < 0.01) but not in the AA condition (27.33 ± 3.60 vs 46.92 ± 10.41, *p* = 0.057). There was no significant difference between the two conditions for the changes in plasma ACTH during exercise (19.59 [− 0.7, 39.8] vs 28.96 [13.5, 44.4], *p* = 0.454). ANOVA for the cortisol/ACTH ratio showed a significant main effect for time (*p* < 0.01), with post hoc t-tests revealing significant differences between before and after exercise in both conditions (*p* < 0.01). The changes in the cortisol/ACTH ratio before and after exercise were not significantly different between the two conditions. The correlations between the changes in cortisol and ACTH are displayed in Fig. 3. Strong correlations were found between the level of cortisol and ACTH in both the AA condition (*r* = 0.58, *p* < 0.01) and the placebo condition (*r* = 0.95, *p* < 0.01), and the regression lines of the relationship between cortisol and ACTH in the AA condition and the placebo condition were almost equal in the study.

### Biological parameters

The effects of supplementation with the AA mixture on the other biological parameters are shown in Table 2. A two-way repeated measures ANOVA (treatment × time) for blood glucose, plasma lactate, plasma ammonia, serum CPK, serum total ketone body, and serum free fatty acid revealed significant main effects for time (*p* < 0.01). Post hoc t-tests showed that the level of blood glucose after exercise was significantly lower than that before exercise in both conditions (*p* < 0.01) and that the other parameters significantly increased during exercise in both conditions (*p* < 0.01). No significant differences were observed between the two conditions for the changes in these parameters during exercise.

**Table 2:** Effects of the supplementation with the AA mixture on blood biological parameters

		Before exercise	After exercise		Change between before and after exercise (95% CI)
Blood glucose (mg/dL)	AA	82.1 ± 2.4	76.1 ± 2.6	##	−6.1 (− 9.8, − 2.3)
	Placebo	79.5 ± 2.0	73.8 ± 1.8	##	−5.7 (− 9.5, − 1.8)
Plasma lactate (mg/dL)	AA	9.3 ± 1.1	18.4 ± 2.2	##	9.1 (5.5, 12.6)
	Placebo	10.1 ± 1.3	17.5 ± 2.5	##	7.5 (2.3, 12.6)
Plasma ammonia (μg/dL)	AA	50.5 ± 3.6	104.4 ± 9.4	##	53.9 (31.8, 76.0)
	Placebo	46.6 ± 2.2	99.7 ± 6.9	##	53.1 (39.9, 66.4)
Serum CPK (U/L)	AA	120.5 ± 8.2	140.3 ± 8.9	##	19.8 (15.7, 23.9)
	Placebo	116.5 ± 9.2	136.3 ± 10.1	##	19.9 (15.5, 24.3)
Serum total ketone body (μmol/L)	AA	40.2 ± 5.3	145.9 ± 27.1	##	105.7 (50.3, 161.1)
	Placebo	38.4 ± 4.8	161.0 ± 32.5	##	122.6 (51.9, 193.4)
Serum free fatty acid (mEq/L)	AA	0.26 ± 0.03	1.03 ± 0.08	##	0.77 (0.62, 0.93)
	Placebo	0.21 ± 0.03	1.02 ± 0.12	##	0.81 (0.57, 1.05)

Values are the mean ± SEM or change score with 95% CI (*n* = 15). ## *p* < 0.01 compared to before exercise

## Discussion

The present study demonstrated that supplementation with the AA mixture containing arginine, valine, and serine suppressed the exercise-induced increase in plasma cortisol, which supported our hypothesis that AA mixture supplementation would suppress the cortisol response during exercise.

Cortisol is released by exercise-induced stress [3–6] in response to glycogen depletion or reduced blood glucose levels to delay fatigue, which is also reported to increase proteolysis [12–15]. In this study, prolonged exercise significantly increased the level of cortisol in the placebo condition, which demonstrated that the exercise condition was high enough to induce cortisol release in the study subjects. In contrast, the level of cortisol did not change in the AA condition, and there was a significant difference between the AA condition and the placebo condition in the cortisol concentration after exercise. These results indicated that acute supplementation with the AA mixture significantly suppressed the increase in cortisol during prolonged exercise, which suggested that the AA mixture might contribute to less stress or better physical condition during exercise and the reduction in muscle protein breakdown after exercise.

ACTH is known as one of the factors involved in the stress response of the HPA axis. ACTH secreted from the pituitary induces cortisol release from the adrenal gland [1, 2]. In the present study, the level of ACTH was significantly elevated by exercise in the placebo condition, which indicated that the cortisol increase in the placebo condition was largely caused by the increased ACTH release. In the AA condition, while no significant differences were observed between before and after exercise on the ACTH concentration, there was a trend toward an increased ACTH level between before and after exercise. In addition, there were no significant differences in the level of ACTH between the two conditions. These results suggested that AA supplementation might not directly affect ACTH secretion during exercise in this study. There were no significant differences in the cortisol/ACTH ratio, which was used as a measure to assess adrenal responsivity [30–32], between the AA condition and the placebo condition in the study. In addition, the correlations between the changes in cortisol and ACTH were evaluated in the study. In the case that the degree of suppression of cortisol and ACTH release by the AA mixture was different, it was expected that there was a difference in the regression lines between the AA condition and the placebo condition. However, in the present study, the regression lines of the relationship between the levels of cortisol and ACTH in the AA condition and the placebo condition were almost equal, which indicated that the ACTH release from the pituitary and the cortisol release from the adrenal gland were suppressed at almost the same level by the AA mixture supplement. However, because one subject’s data whose values seemed to differ greatly from the other data might have had a certain impact on the generation of the regression lines in the study, further investigations with an increased sample size should be conducted to obtain more accurate findings. Future studies are also needed to clarify the effect of the AA mixture on CRH release from the hypothalamus, the most upstream factor of the HPA axis.

Blood glucose levels are strongly related to cortisol release. Tabata et al. reported that decreases in the blood glucose concentration lead to enhanced secretion of ACTH and cortisol during prolonged exercise in humans [7, 8]. In this study, the blood glucose levels were significantly decreased both in the AA condition and the placebo condition, and there were no significant differences between the two conditions. Thus, the blood glucose level was not related to the cortisol suppressive effect of the AA mixture in the current study. Cortisol breaks down muscle or liver glycogen to maintain the blood glucose level when blood glucose levels decrease [33, 34]. It was reported that acute valine supplementation maintained the liver glycogen content and suppressed the cortisol level in rodents [26]. These findings suggest that muscle or liver glycogen might be maintained by the AA mixture supplement in this study.

Arginine was reported to improve lipid metabolism [23–25]. Lucoti et al. reported that long-term oral arginine treatment (8.3 g/day) decreased fat mass and waist circumference as well as improved glucose metabolism and insulin sensitivity [35]. Hurt et al. reported that 3 g of L-arginine three times a day for 12 weeks could be effective at reducing central adiposity in obese patients [36]. In the present study, there were no significant differences in the levels of serum total ketone body or free fatty acid between the two conditions before and after exercise, which suggested that acute supplementation with the AA mixture containing 1.8 g of arginine did not exert significant effects related to lipid metabolism in this study. The lipid metabolism-related biomarkers were not related to the effect of the AA mixture in the study.

CPK has been used as a marker of muscle cell damage because it is released into blood when a disruption occurs in the sarcomere [37, 38]. It was reported that BCAA or phosphatidylserine supplementation could suppress the increase in the level of CPK after exercise [39, 40]. Ammonia, which is reported to have neurotoxicity, is produced by the catabolism of muscle proteins when energy is depleted during exercise [41, 42]. It was reported that arginine could suppress the increase in the level of ammonia during exercise [43]. In the present study, there were no differences between the two conditions in the levels of CPK and ammonia, which indicated that these biomarkers were not related to the effect of the AA mixture on the level of cortisol.

Although the mechanism of the suppressive effect of the AA mixture could not be clarified in the study, we propose several possibilities for the mechanism of the effect of the AA mixture. First, supplementation with the AA mixture might have contributed to suppressing cortisol release via NO synthesis. Arginine is known as a substrate for nitric oxide (NO) synthesis [44]. It was also reported that NO activity modulates the response of the neuroendocrine component of the HPA axis [45, 46]. Second, the effect of the AA mixture might be caused by the attenuation of serotonin synthesis. It was revealed that serotonin activates the HPA axis via serotonin receptor stimulation [47]. We have also clarified that supplementation with the AA mixture used in the study decreased the tryptophan/BCAA ratio during exercise in a previous study, suggesting that the AA mixture might attenuate the synthesis of serotonin [28]. In addition, the AA mixture might contribute to reducing the cortisol response via phosphatidylserine production, which was reported to attenuate the release of ACTH and cortisol during moderate intensity exercise [27]. It was also demonstrated that oral administration of serine could increase the concentration of serine in the brain and synthesize phosphatidylserine [48, 49].

There are several limitations of the present investigation. We did not control subjects’ diets except for breakfast on the exercise trial day. Although subjects were instructed to consume their usual diets, it is possible that the differences in their energy intake limited the evaluation of the effect of the AA mixture supplement. A diet record analysis should be conducted in future studies. We also did not limit dietary supplements other than asking subjects not to consume any supplements on the trial day. Furthermore, we did not measure the levels of CRH, NO, serotonin, or phosphatidylserine, which might be involved in the mechanism of the effect of the AA mixture in this study. Further examinations measuring the effect of the AA mixture on the production of these candidate factors will support the results of this study.

## Conclusions

In conclusion, this study demonstrated that acute AA mixture supplementation significantly regulated the cortisol response during exercise without altering exercise-related biological parameters such as glucose or lipid metabolism. Although the AA mixture might affect cortisol release through NO or phosphatidylserine synthesis or attenuation of serotonin synthesis in the brain, it is necessary to clarify the detailed mechanism of this process in further investigations.

## Data Availability

The datasets used and/or analyzed in the current study are available from the corresponding author on reasonable request.

## Abbreviations

HPA: Hypothalamic-pituitary-adrenal
CRH: Corticotropin-releasing hormone
ACTH: Adrenocorticotropin
AA: Amino acid
BCAA: Branched-chain amino acid
CPK: Creatine phosphokinase
SEM: Standard error of the mean
CI: Confidence interval
ANOVA: Analysis of variance
